# For those with schizophrenia, does cannabidiol make cannabis use safer?

**DOI:** 10.1038/s41386-025-02199-9

**Published:** 2025-08-26

**Authors:** Anya K. Bershad

**Affiliations:** 1 Department of Psychiatry and Biobehavioral Sciences, Jane and Terry Semel Institute for Neuroscience and Human Behavior, UCLA, Los Angeles, CA USA; 2 VA Greater Los Angeles Healthcare System, Los Angeles, CA USA

**Keywords:** Schizophrenia, Drug development

Anecdotal reports of cannabis users suggest that one component of cannabis, cannabidiol (CBD), ameliorates many of the adverse effects of cannabis. For individuals with schizophrenia, there is some evidence that CBD given alone (without other components of cannabis) can reduce psychotic symptoms [1]. Patients sometimes ask whether they can reduce their risk of symptom exacerbations by seeking out cannabis products with a high ratio of CBD to tetrahydrocannabinol (THC). Indeed, it would be an elegant story if one of the components of cannabis could treat symptoms induced by another component. A new study addresses this question for the first time.

Chesney et al. [2] report the results of a placebo-controlled trial with a crossover design in which individuals with schizophrenia or schizoaffective disorder with comorbid cannabis use disorder (*N* = 30) were randomized to receive either oral CBD (1000 mg) or placebo 3 h before inhaling vaporized cannabis. They measured delayed verbal recall using the Hopkins Verbal Learning Test-revised and psychotic symptoms using the Positive and Negative Syndrome Scale (PANSS). The authors hypothesized that pretreatment with CBD would ameliorate the negative effects of cannabis on delayed verbal recall and the effects of the drug on positive symptoms.

The results of this study were surprising and directly contradicted the authors’ hypotheses. First, the authors reported that CBD worsened the effects of cannabis on delayed verbal recall, their primary outcome measure. They reported that CBD pretreatment was also associated with a greater increase in positive symptoms on the PANSS. CBD did not lead to significant differences in subjective drug effect ratings of “feel drug”, though it did lead to greater increases in systolic blood pressure.

The authors also collected plasma levels of CBD, THC, and their metabolites. They reported that CBD pretreatment did not increase exposure to the active metabolite of THC, 11-hydroxy-THC, but it did increase exposure to the inactive metabolite, 11-carboxy-THC. Further, the AUC for plasma CBD levels was positively correlated with the change in both delayed recall and PANSS positive symptoms. This correlation lends further support to the validity of the surprising finding that CBD pretreatment worsened the effects of THC on cognition and positive symptoms.

While the results of this study were unexpected, the elegant study design allows us to have confidence in the findings. The study population was carefully selected to have diagnoses of both schizophrenia and cannabis use disorder, rather than schizophrenia alone. While this decision necessitated addressing the issue of tolerance, it is the population for whom the question of the effects of CBD on cannabis use is most pressing. Second, the authors used an ecologically valid route of administration of cannabis (inhalation) and accounted for tolerance to cannabis by including repeat dosing sessions for participants who did not have a significant response to lower doses. Finally, plasma levels of the compounds and their metabolites were obtained, allowing for the investigation of pharmacokinetic explanations for the findings.

This well-designed study represents the first placebo-controlled investigation of the effects of pre-treatment with CBD on cannabis-induced cognitive impairment and psychotic symptoms in individuals with schizophrenia and cannabis use disorder. The effects of CBD on adverse responses to cannabis have been previously investigated in healthy volunteers by the authors of this article and others. Results of these studies are mixed, with some showing CBD ameliorates these effects [3], some showing no effect [4], and some showing results consistent with the findings reported here, that CBD exacerbates these effects [5]. The studies reporting that CBD exacerbates THC’s effects attributed the finding to the inhibition of hepatic metabolism of THC by CBD. However, Chesney et al. assessed plasma levels of CBD, THC, and their metabolites to test this and reported that their findings cannot be explained by such a pharmacokinetic interaction. What are we to make of these unexpected findings?

This study inspires many more questions about the relationship between CBD, cannabis use, and psychotic symptoms. Since this is the first study of this nature to be conducted in either individuals with schizophrenia, or individuals with cannabis use disorder, one question that arises is whether individuals in either category have a different response to CBD (as it relates to cannabis effects) than do healthy controls. As the authors note, individuals with schizophrenia and cannabis use disorder both have alterations in the endocannabinoid system that may affect responses to CBD. Since CBD may have some effect as an adjunct to antipsychotic medications, it would be helpful to know whether individuals with comorbid cannabis use disorder and schizophrenia may still benefit from CBD for baseline psychotic symptoms, if not from CBD for cannabis-induced symptoms. A further consideration is that CBD was given orally in this study, and a separate, but related, question is whether vaporized cannabis with different ratios of CBD to THC would produce similar effects.

Ultimately, this study shows us that oral CBD, when used prior to cannabis, can worsen cannabis-induced symptoms for individuals with schizophrenia. So what should we tell our patients with schizophrenia who are using cannabis and interested in CBD? To begin with, cannabis use is particularly dangerous for those with psychotic illnesses, as it can exacerbate symptoms like paranoia and cognitive impairment. Given these new findings, we should let them know that CBD probably won’t protect them from cannabis-related harms—and might even make things worse. For now, a clear discussion of the risks associated with cannabis use is the best we can offer.
